# Polymer-Based Functional Cantilevers Integrated with Interdigitated Electrode Arrays—A Novel Platform for Cardiac Sensing

**DOI:** 10.3390/mi11040450

**Published:** 2020-04-24

**Authors:** Pooja P. Kanade, Nomin-Erdene Oyunbaatar, Dong-Weon Lee

**Affiliations:** 1 MEMS and Nanotechnology Laboratory, School of Mechanical Systems Engineering, Chonnam National University, Gwangju-61186, Korea; 2 Center for Next Generation Sensor Research and Development, Chonnam National University, Gwangju-61186, Korea

**Keywords:** SU-8 cantilever, contraction force, electrochemical impedance spectroscopy, cardiomyocytes, Verapamil, E-4031

## Abstract

Heart related ailments are some of the most common causes for death in the world, and some of the causes are cardiac toxicity due to drugs. Several platforms have been developed in this regard over the years that can measure electrical or mechanical behavior of cardiomyocytes. In this study, we have demonstrated a biomedical device that can simultaneously measure electrophysiology and contraction force of cardiomyocytes. This dual-function device is composed of a photosensitive polymer-based cantilever, with a pair of metal-based interdigitated electrodes on its surface, such that the cantilever can measure the contraction force of cardiomyocytes and the electrodes can measure the impedance between cells and substrate. The cantilever is patterned with microgrooves so that the cardiomyocytes can align to the cantilever in order to make a higher cantilever deflection in response to contraction force. Preliminary experimental results have identified the potential for use in the drug-induced cardiac toxicity tests, and further optimization is desirable to extend the technique to various bio-sensor areas.

## 1. Introduction

Heart related ailments are some of the most common causes for death in the world, and some of the causes are cardiac toxicity due to drugs. Cardiac toxicity is one of the major factors affecting success in drug tests in clinical studies [1]. In total, 90% of drugs that enter clinical trials fail to commercialize owing to their toxic side effects on the heart [2]. Hence, development of such techniques that can measure electrophysiology and contraction force of cardiomyocytes is of critical importance. Various techniques have been proposed till date that can measure these parameters. The patch clamp technique is an established technique to measure electrophysiology of the cells, however it is an invasive technique and data of only a single cell can be obtained. It is also an expensive technique and requires high amount of expertise [3,4]. Hence, a high throughput technique is required to reduce costs and time in the early stage of drug development. Over the years, several techniques have been developed to enhance the efficiency of the analyzing methods. In addition to conventional electrophysiological methods, many researches have become interested in measuring the change in contractile force of cardiomyocytes. In order to measure mechanical response in the form of contraction force, micro-posts and cantilevers have been used, such that these devices would measure the amount of deflection occurring because of the cardiomyocytes and contraction force can be calculated based on this deflection [5,6,7,8]. Flexible polydimethylsiloxane (PDMS) micro-posts have been fabricated with microgrooves to measure the contraction force of cardiomyocytes [5]. However, it is difficult to get real time information at the tissue level using this technique. So, to measure the contraction force, technique of measuring cantilever deflection is a better and efficient technique in which real time data and beating pattern can also be obtained. SU-8 cantilever arrays have been developed for preliminary screening of cardiac toxicity due to drugs [9,10]. PDMS cantilever integrated with piezo-resistive sensor on its surface have also been developed to measure the contractile behavior of cardiomyocytes [11,12].

Measurement of electrical cell substrate impedance spectroscopy (ECIS) of cells is of equal importance, since information that cannot be obtained from mechanical response of cells, such as information related to cell adhesion, number of cells, growth and cell-substrate interaction, can be obtained from ECIS [13]. There have been various reports of ECIS being used to measure properties of various kinds of cells [14,15,16], including cardiomyocytes [17,18,19]. ECIS is a non-invasive and label-free method and real-time information on cell behavior can be obtained. Interdigitated electrode array (IDE) is a two-electrode arrangement for measuring impedance that have been extensively used to measure properties of cardiomyocytes [20], since they have a distinct advantage of having a relatively simple geometry and higher sensitivity. Commercially available impedance measuring systems are also in place, such as xCELLigence RTCA Cardio system [21,22,23,24]. IDEs have also been fabricated in conjunction with circular microelectrode arrays (MEAs) to measure extracellular field potential along with impedance [18,25,26].

However, till date, measurement of impedance using a pair of IDEs and contraction force with the help of a cantilever—thereby measuring interaction of cells with substrate and contraction force simultaneously—has not been done. It is important to understand the adhesion characteristics of cardiomyocytes along with their beating behavior and how the adhesion changes when the cells grow. Our team had previously proposed a device that integrated measurement of contraction force with electrophysiology [27]. Impedance was measured using a set of MEAs and contraction force was measured using the displacement of a cantilever. However, impedance measurement using MEAs has its limitations since the inter-electrode distance is too high to obtain accurate impedance results of the overall cells. In addition to it, resistance of the bulk solution would be the dominant factor in the impedance result. In this work, we propose a device wherein impedance is measured with the help of IDEs. The IDE has been fabricated on the top of a cantilever. While IDEs, like other two-electrode methods for measuring impedance, can be sensitive to electrode polarization impedance, the electrodes have been optimized in such a way as to minimize the polarization impedance. The electrode polarization impedance is more prominent where electrode area is small, causing relevant impedance contributions of the system to be masked at lower frequencies. However, in our case, the electrode area is sufficiently large, so the electrode polarization impedance effect did not affect the data. The cantilever-cum-IDE surface has been patterned with microgrooves. Multiple devices have been fabricated with varying electrode dimensions. The IDE is fabricated with gold as electrodes on the photosensitive polymer base layer of cantilever. Microgrooves are also patterned with the same photosensitive polymer material. Neonatal rats’ cardiomyocytes were cultured on the device for analyzing the bending displacement of cantilever and impedance from IDE. Further, to check the response to cardiac toxicity, two drugs—namely Verapamil and E-4031—were added and the cantilever deflection and IDE impedance was measured.

## 2. Working Principle of the Device

**Figure 1:**
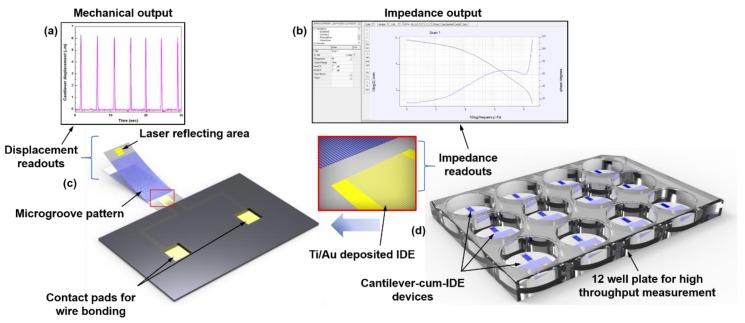
Schematic of the device and its working principle. (**a**) Cantilever displacement measured by the laser vibrometer with the help of laser reflecting area at the end of the cantilever. Output can be viewed in LabVIEW (version 9.0), (**b**) impedance spectrum measured from interdigitated electrode array (IDE) with the help of IviumStat software, (**c**) schematic of the cantilever device denoting positions of IDEs on the cantilever, microgrooves, laser reflecting area and contact pads for wire bonding, (**d**) schematic of the cantilever devices in a 12 well culture plate for high throughput measurement.

This device can measure impedance of cardiomyocytes and their contraction force simultaneously in real time. We can understand some of the key characteristics of cells with the help of impedance, such as growth and adhesion. Upon cardiomyocyte isolation on this device, impedance was measured using IDEs fabricated on top of a miniature polymer cantilever. The cantilever displaces from its initial position when the cells contract, using which contraction force of the cells was measured. Microgrooves are patterned on the surface of the cantilever as guiding layer to enhance maturation and promote self-organization of the cardiomyocytes. Microgrooves are known to maximize the contraction force of cardiomyocytes [27]. Figure 1a shows the schematic of this IDE-fabricated-on-cantilever device. As shown in the schematic, microgrooves are patterned on the entire area of the cantilever, except the laser reflecting area and IDE pattern. The laser reflecting area should have high reflectivity so that the laser vibrometer senses the reflected light. And in case of IDEs, in order to maintain the contact between cardiomyocytes and the IDE pattern, microgrooves are avoided.

ECIS is an effective tool to monitor growth and cytotoxicity of cardiomyocytes. To monitor the cell impedance, a sinusoidal voltage generated by the impedance measuring instrument is applied onto the IDEs, and the output impedance is recorded by data acquisition module. The impedance Z_0_ is set as baseline before cell seeding. The impedance will increase when cells are cultured on the sensor surface, which impeded the ion current between IDEs. Generally, the impedance value is normalized by cell index (CI) value, which reflected the ratio of the cell induced impedance changes ΔZ to baseline impedance Z_0_ [18]. The CI value is an arbitrary unit which reflects the cell number, morphology, and attachment. ECIS is non-invasive recording technology.

To measure the simultaneous cantilever displacement, a uniform metallic pattern is deposited on the far edge of the cantilever, known as the laser reflecting area. Contraction force of cardiomyocytes has been measured with the help of the cantilever. Cardiomyocytes seeded on the cantilever will start contracting as they grow, hence resulting in the displacement of the cantilever. This displacement is measured using a laser vibrometer, that will measure displacement of the laser reflecting area. This instrument uses a position-sensitive photodiode sensor as a laser that is irradiated on the reflective plate of the cantilever. If the laser is aligned perpendicularly to surface of the cantilever, the vibrometer measures the out-of-plane vibration to the surface. The vibrometer measures the amount of vibrations at a single point on the surface and records the vibration characteristics as a function of time. Figure 1b shows the working methodology of the device of simultaneous measurement of cantilever displacement and impedance.

## 3. Methods and Materials

### 3.1. Device Fabrication

**Figure 2:**
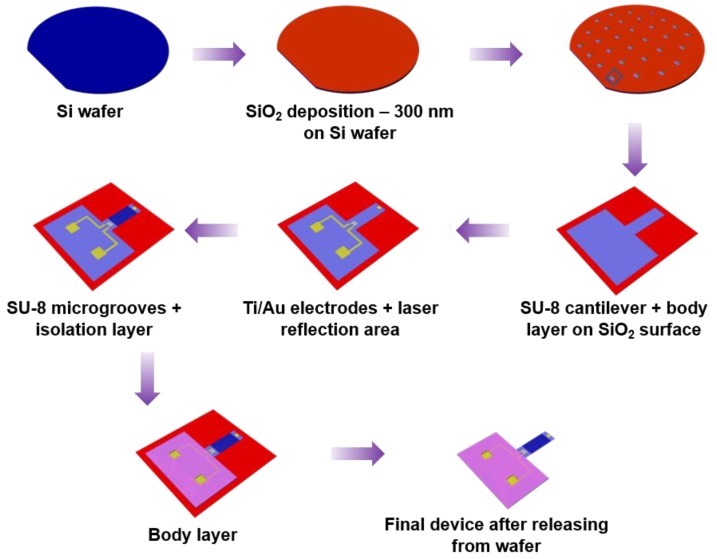
Fabrication process flow of the dual function device.

Figure 2 shows the process flow steps for device fabrication. Silicon (100) wafer first underwent thermal oxidation (wet oxidation) and a 300 nm thick sacrificial silicon dioxide (SiO_2_) layer was formed on the wafer. On this, the functional cantilever device was fabricated by photolithography techniques. The first pattern of cantilever was defined using the polymer photoresist SU-8 3010. The thickness of the fabricated cantilever is ~15 µm. The length and width of the cantilever are 6 mm × 2 mm. Next, the Ti/Au metal layer of thickness 10/100 nm was deposited by electron beam evaporation technique. The IDE pattern and the laser reflecting area on the cantilever were formed by wet-etching of the Ti/Au layer. Next, microgrooves of width 3 µm, spacing 3 µm and thickness of 1 µm were fabricated using SU-8 2002. Along with microgrooves, SU-8 was also deposited on the legs of IDE in order to isolate them from cardiomyocytes and cell culture medium. The last pattern is the body layer of the device, that was fabricated to give strength to the device. Thickness of this layer is ~100 µm. Lastly, the cantilever device was released from SiO_2_ layer using buffered oxide etchant (BOE) solution and the devices were released from the wafer. Figure S1a shows the optical image of the cantilever device along with IDE and Figure S1b shows the microgroove pattern.

Three types of IDEs were fabricated, keeping in view that the cell constant should be low. The details of the IDE dimensions are given in Table S1. Optical image of the three IDE patterns are shown in Figure S1c–e.

The devices were sterilized first under ultraviolet light for 400 s, and then baked at 95 °C for 5–6 h. In order to prepare the device for cell seeding in the well plate, contact wires were first connected to the contact pads of IDE using a conductive epoxy and cured at 80 °C. This conductive epoxy was then isolated from the cell culture medium using polydimethylsiloxane (PDMS).

### 3.2. Cell Culture

All animal experiments were approved by the Animal Ethics Committee of Chonnam National University. Ventricles were harvested from one-to-three day old neonatal Sprague-Dawley rats. For digesting cardiomyocytes from the ventricular tissue, the ventricular tissue was digested with a mixture of 0.4 mg·mL^−1^ collagenase and 0.6 mg·mL^−1^ pancreatin solution. Then the digested solution was divided into cardiomyocytes and fibroblast layers using the percoll, and the separated layers pre-plated in order to prepare high purity cardiomyocytes.

Cardiomyocytes were cultured on the device including the cantilever and IDE at a density of 1000 cardiomyocytes per mm^2^ in a culture solution that comprised of 67% Dulbecco’s modified Eagle medium (DMEM, LONZA, Gyeongsan-si, Korea), 17% Heparin sodium salt from porcine intestinal mucosa (M199, Sigma-Aldrich, Gyeongsan-si, Korea), 10% Horse serum (HS, Sigma-Aldrich, Gyeongsan-si, Korea), 5% fetal bovine serum (FBS, Sigma-Aldrich, Gyeongsan-si, Korea) and 1% penicillin streptomycin (P/S, Sigma-Aldrich, Gyeongsan-si, Korea). The cardiomyocytes were cultivated in an incubator that maintained the temperature at 37 °C and carbon dioxide (CO_2_) at 5%. The devices were immersed in 2 mL of culture solution each. The culture solution was replaced every three days.

### 3.3. Simultaneous Measurement of Impedance and Cantilever Displacement

EIS was measured using the impedance workstation IVIUM Technologies CompactStat (Standard type, Eindhoven, The Netherlands) instrument in the constant potential mode. Impedance spectrum was measured at a constant AC signal of 150 mV in the frequency range of 100 Hz to 2 MHz. We used the two-electrode system to record impedance.

A laser vibrometer-based measurement system was used in order to measure the contraction force of cardiomyocytes on the cantilever. Minimum displacement that the laser vibrometer can measure is ~120 nm. Hence, the laser vibrometer is sensitive enough to measure the synchronized contraction force of cardiomyocytes, that results in the displacement of the cantilever [9]. The experimental setup consisted of a laser vibrometer (Polytec GmbH) that measured displacements in nanoscale and a home-made stage-top incubator that maintained the temperature of a well plate. The system can measure the cantilever displacement precisely by irradiating a laser on the reflective area on the far side of the cantilever. The laser vibrometer is controlled via LabVIEW (version 9.0).

Impedance of samples was initially measured before cell seeding in the cell culture medium. Thereafter, in order to measure the adhesion of cells with substrate, impedance was measured every 3 h, up to 72 h. On day 4 after cell seeding, cell beating was observed and hence, cantilever displacement could also be measured. From day 4, cantilever displacement and impedance were measured simultaneously. Cell culture medium was refreshed once every three days.

On day 7, the samples were subjected to drug toxicity screening. Effect of two drugs, namely Verapamil and E-4031, were tested on the device in order to understand the response of our device to cardiac toxicity. Verapamil drug, which is a calcium ion channel blocker, was added of concentrations 150 nmol/L, 300 nmol/L, 500 nmol/L and 1000 nmol/L, and simultaneous impedance and cantilever displacement were measured for up to 24 h for each concentration. E-4031, which is a potassium ion channel blocker of the hERG channel, was added in concentrations of 5 nmol/L, 10 nmol/L, 20 nmol/L and 30 nmol/L and impedance and cantilever displacement were measured simultaneously for up to 24 h.

### 3.4. Immunocytochemistry (ICC) Analysis for Cardiac Marker

Immunocytochemical staining of the cultured cardiomyocytes was performed using the following antibodies. Using a typical process, the cardiomyocytes were placed in a 4% formaldehyde-dissolved Dulbecco’s phosphate-buffered saline (DPBS) solution for 20 min at room temperature and, then, washed three times with DPBS. Then, the cardiomyocytes were permeabilized with 0.1% Triton X-100 (Sigma–Aldrich) in DPBS for 5 min and blocked for 30 min in 3% bovine serum albumin (BSA) (Sigma–Aldrich). Next, the cardiomyocytes were incubated with the primary antibodies, including mouse monoclonal α-sarcomere actinin (Abcam), and Troponin-T (TnT) (Abcam), which were diluted to a ratio of 1:100 with 1% BSA solution, for 90 min at room temperature. The secondary antibodies (Alexa Fluor 488 Goat anti-mouse lgG conjugate and Alexa Fluor 568 Goat anti-rabbit lgG+ (H+L) conjugate) were diluted to 1:200 in the same blocking solution that was used for the primary antibodies and the cardiomyocytes were incubated in them for 90 min at room temperature. Finally, the samples were mounted using coverslips and ProLong Gold Antifade (Sigma–Aldrich). Finally, the cardiomyocytes were analyzed by inverted confocal laser scanning microscopy (Leica TCS SP5 XCLSM, Germany).

## 4. Results and Discussion

As mentioned earlier, IDEs of three different dimensions were fabricated, the details of which are mentioned in Table S1. The final devices in cell culture medium are shown in Figure S2a. The connecting wires extending from each device are used to measure impedance. 

During design of the IDEs, it was kept in mind that the cell constant (K) should be kept low. A low cell constant ensures higher sensitivity of the device in response to cardiomyocyte changes. For simple geometries like ours, theoretical value of cell constant (K) can be calculated by [22]:

$$K=2\frac{\sqrt[{3}]{S/W}}{L\left(N-1\right)}$$

where S is the spacing between electrodes, W is the electrode width, L is the electrode length, and N is the number of fingers. In our case, the cell constant values are very near to each other and less than 1. Hence, our IDEs have sufficiently low K value to be used for impedance measurement of cardiomyocytes.

In order to measure impedance and the corresponding change with dimensions and surface area, base impedance of each IDE was measured and compared. The base impedances over the spectrum of frequencies from 100 Hz to 2 MHz are shown in Figure S2b. Type 1 IDE showed the highest impedance, followed by type 2 and the least was type 3. A lower base impedance is desirable so that minute changes in impedance can also be detected. Hence, in the present work, the types 2 and 3 IDE designs have been used, and all the data for further analysis have been normalized.

**Figure 3:**
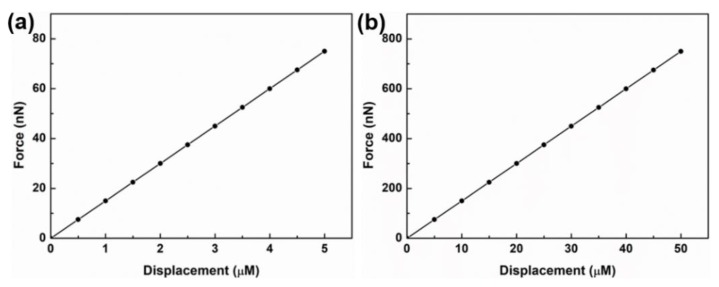
(**a**,**b**) Measurement of force as a function of cantilever displacement.

The next step is to characterize the contraction force experienced by the cantilever. As is well known, the contraction force generated by cardiomyocytes is so small that the cantilever needs to be highly sensitive in order to measure the force. In our case, first the displacement experienced by the cantilever is first measured, and then convert it to force by using the calculated value of spring constant. Spring constant of our cantilever (6 mm × 2 mm × 15 µm) is 0.015 N/m. As we can see from Figure 3, the force changes almost linearly with respect to cantilever displacement. When an external force is applied to a cantilever and it undergoes displacement, a part of the cantilever on one side of the neutral axis undergoes compression while the other part undergoes tension. The neutral axis is the axis where there is no effect of applied force. The data obtained experimentally demonstrate that our device exhibits wide range of detection limits ranging from 7.5 nN for 500 nm displacement, and 750 nN for 50 µm displacement. This shows that this device is sensitive enough to detect even smaller variation in the displacement of the cantilever caused by contractile force of the cardiomyocytes.

Further, when the cantilever oscillates, it exerts some amount of strain on every portion of the cantilever. The area near fixed end of cantilever experiences the highest strain, and this is the area near which the IDEs have been placed. So, in order to see if there might be any influence of the experienced strain on our electrodes, minute displacements to the cantilever, ranging from 10 µm to 500 µm were applied, and corresponding impedance spectrum was recorded at each step. Figure S3 shows output of the measured data. As we can see, the impedance spectra at all the displacements is identical at all the frequencies. Hence, we can say that during our experiment of cultured cardiomyocytes on the device, there has been no influence of the strain exerted on the impedance readouts at any frequency, and both cantilever displacement and impedance have been measured independently.

**Figure 4:**
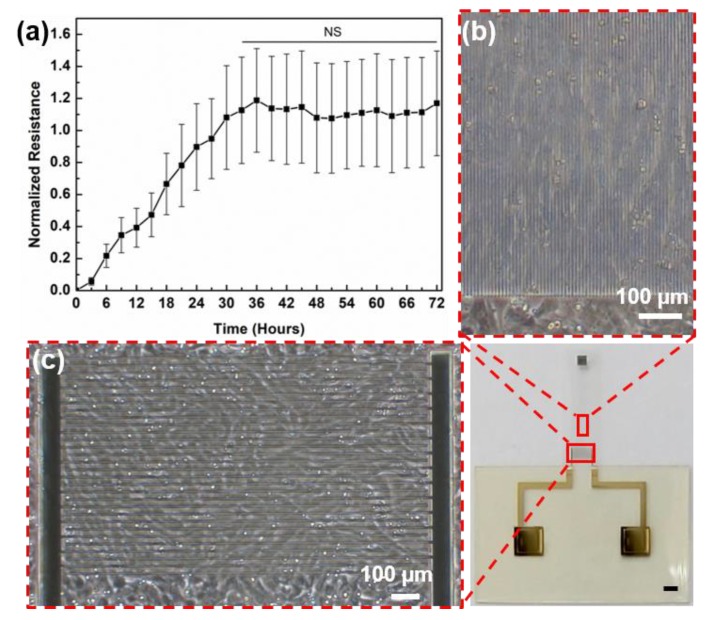
(**a**) Normalized resistance measured for up to 72 h, (**b**) Optical image of distribution of cardiomyocytes on microgroove-patterned cantilever and (**c**) on the IDEs. Scale bar on the device image is 1 mm.

Upon confirmation of the above readouts, our device was ready for cardiomyocyte seeding. Our next objective was to understand how the cells adhere to the surface from the time of cell seeding. For this, first the base impedance of the cell culture medium was measured before cell seeding. Then after cell seeding, impedance was measured for up to 72 h. Figure 4a shows the graph of normalized resistance versus time measured up to 72 h. It should be noted that the graph shows normalized resistance, which is extracted from the impedance data, since information pertaining to adhesion between cells and substrate can be extracted from change in resistance [19]. The graph shows normalized resistance at a frequency of 5 kHz. This frequency was chosen based on the impedance change observed after cell seeding, as shown in Figure S4. The change in impedance 12 h after cell seeding was more prominent in the frequency range from ~400 Hz to ~ 50 kHz. Hence, this frequency was chosen during data plotting. The inset of Figure S4 shows the equivalent circuit of the system formed from the two-electrode arrangement of impedance analysis. Here, R_S_ is the series resistance consisting of the solution resistance, electrode resistance and connector resistance, Z_CPE_ is the electric double layer capacitance between electrode and cell culture medium, and R_P_ and C_P_ are the resistance and capacitance between cardiomyocytes and electrodes.

As can be seen from Figure 4a, resistance is almost linearly increasing from 0 to 36 h, after which it is starting to stabilize. At 36 h, resistance reached 118% of its base value, after which it began to saturate and remained stable at nearly 113% of its of its initial value. This is in agreement with previously reported work [21]. Based on this data, we can see that cells start to grow and adhere to the substrate soon after they are seeded and by 36 h, properly adhere to the substrate. The standard deviation (SD) of the normalized resistance in the adhesion profile is high after 18 h. This is because although the experiment was performed on three biologically independent samples, the number of cells and their growth characteristics vary in each sample. This will also affect the number of layers of cardiomyocytes attached forming on the substrate. This in turn affects the change in resistance values, which can be different in different samples—finally affecting the normalized values.

Figure 4b,c show the distribution of cardiomyocytes on the IDE and on the microgroove-patterned cantilever respectively. We can see that cardiomyocytes are evenly distributed on the IDE as well as cantilever. The optical image indicate that the cells could grow along with the microgrooves and prove the validity of facilitating the alignment of cardiomyocytes through the longitudinally patterned microgrooves [24]. The images were taken on day 4 after cell seeding.

**Figure 5:**
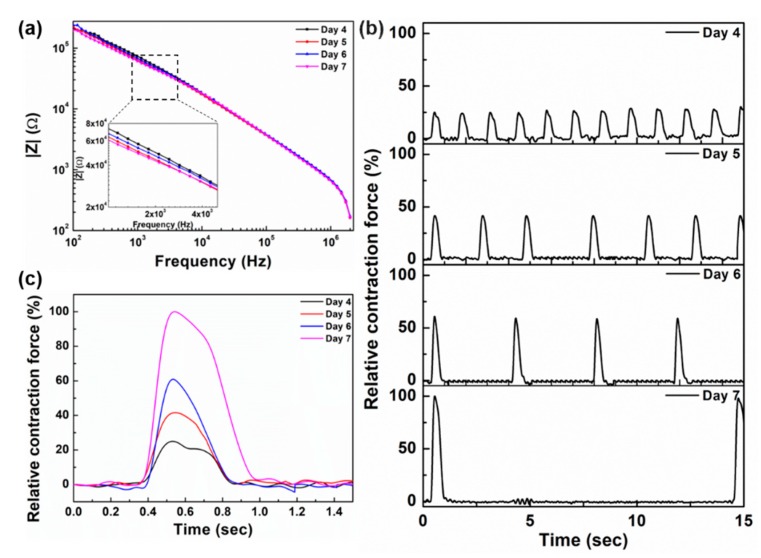
(**a**) Bode plot of the impedance spectrum measured from day 4 to day 7 after cell seeding, (**b**) normalized contraction force from day 4 to day 7 after cell seeding, (**c**) overlapped curves of contraction force from day 4 to day 7.

From day 4 after cell seeding, we began to observe clear cell contraction and resultant cantilever displacement under the microscope and started to simultaneously measure impedance and cantilever displacement. Figure 5a shows the bode plot of the impedance spectrum from day 4 to day 7 and Figure 5b shows the relative contraction force measured simultaneously with impedance. The impedance is remaining nearly the same, indicating that the cells have properly adhered to the substrate, and even if the contraction force changes, it has no direct effect on adhesion. Based on the Bode plot, the values of R_S_, Z_CPE_, R_P_ and C_P_ are 5.177 ± 1.21 kΩ, 5.01 ± 0.85 × 10^−8^ (n = 0.705 ± 0.02), 40.52 ± 7.44 kΩ and 3.29 ± 0.56 nF respectively.

The contraction force of cardiomyocytes is increasing from day 4 to day 7 and consequently the cantilever displacement increases, as can be seen from Figure 5b. The cardiac contraction force increased by 166.4% on day 5 as compared to day 4, 243.56% on day 6 and by 400% on day 7 as compared to day 4. Figure 5c shows the overlapped beating patterns of each day. Along with contraction force, the beating duration of cells also increases each day. Once the cardiomyocytes are cultured on the substrate, they start adhering to the substrate and grow and multiply. Cardiomyocytes get synchronized by day 4 after cell culture, and the contraction force can be measured by the cantilever displacement. After day 4, cardiomyocytes further grow and mature, because of which the contraction force increases, thus resulting in higher cantilever displacement. On day 4, the beating frequency is higher, but as the cells mature, beating frequency starts to decrease, as can be seen from Figure 5b [9].

**Figure 6:**
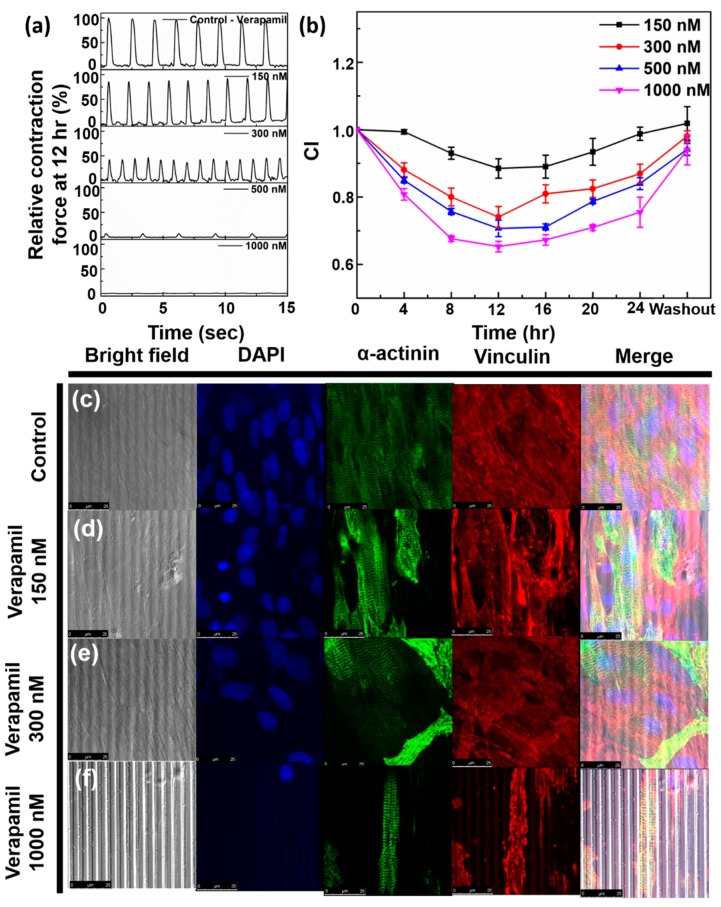
(**a**) Normalized contraction force on addition of Verapamil of concentrations 150 nmol/L, 300 nmol/L, 500 nmol/L, 1000 nmol/L after 12 h, (**b**) Normalized impedance output on addition of Verapamil of concentrations 150 nmol/L, 300 nmol/L, 500 nmol/L, 1000 nmol/L up to 24 h, (**c**–**f**) Immunocytochemistry (ICC) staining data for (**c**) control, (**d**) 150 nmol/L, (**e**) 300 nmol/L, (**f**) 1000 nmol/L Verapamil. Scale bar = 25 µm.

Subsequently, this device was tested for its response to drug toxicity on cardiomyocytes. Firstly, Verapamil, which is a calcium ion channel blocker, was chosen. Verapamil is used to treat cardiac arrhythmia, hypertension and vasodilator during cryopreservation of blood vessels [21]. Different concentrations of Verapamil—namely 150 nmol/L, 300 nmol/L, 500 nmol/L and 1000 nmol/L—were added on different samples and the change in impedance and cantilever displacement were monitored up to 24 h. Figure S5a–d show the output of the change in contraction force at each concentration at various intervals, and Figure 6a shows the relative change in contraction force after 12 h of addition of the drug. It is to be noted that the graphs shown in Figure 6a are of different samples and shown only for reference. Figure 6b shows the output of normalized impedance measured up to 24 h. On addition of 150 nmol/L Verapamil, contraction force decreases slightly initially, but then regains the amplitude within 8 h. Furthermore, the cell index goes down to up to 82% in h, and then starts to recover. In case of 300 nmol/L Verapamil, contraction force decreased to nearly 45% of its current value in 12 h after which it started to recover. It recovered nearly 51% of its base value in 24 h. On the other hand, the cell index at 300 nmol/L decreases to 74% of its base value in 12 h, after which it shows time dependent recovery. In case of 500 nmol/L Verapamil, contraction force came down to 1.5% of its base value within 4 h; however, it started to self-recover in 12 h. Simultaneous measurement of impedance showed that the cell index decreased up to nearly 70% in 12 h, which also began its time-dependent recovery when measured at 16 h. Lastly, when a large concentration of 1 µmol/L Verapamil was added, contraction force reduced to ~0% for the first 14 h, as can be seen from Figure 6d. No beating could be observed from the cardiomyocytes in this period. However, when measured at 14 h, contraction force started showing a few spikes of nearly 1.5% of the base value. At 24 h, 5.5% of the contraction force showed recovery. Whereas the cell index reached a minimum of 65% at 12 h, after which it recovered up to 75% of the base value.

The chronotropic effect of Verapamil can be clearly observed from Figure S5, followed by time-dependent and dose-dependent recoveries of contraction force and cell index (Figure 6b). This readout is in line with the previous reported result [21]. Verapamil reduces calcium influx to prolong atrioventricular node effective refractory period, and slows down the conduction rate, in order to reduce ventricular rate of patients with chronic atrial fibrillation, atrial flutter and paroxysmal supraventricular tachycardia frequency. Verapamil serves as a calcium antagonist that makes pharmacological effects by regulating calcium influx on the plasma of myocardial conduction cells, myocardial contractile cells and vascular smooth muscle cells, without changing the serum calcium concentration.

The obtained data is further justified by immunocytochemistry (ICC) staining experiment. Figure 6c–f shows the bright-field, DAPI (blue), α-sarcomere actinin (green), Vinculin (red) staining and merged images for control, 150 nmol/L, 300 nmol/L and 1000 nmol/L concentrations of Verapamil. The ICC staining experiments were performed 12 h after addition of the drugs. The number of cells gradually decrease from control to 150 nmol/L and to 300 nmol/L, and significantly decrease in case of 1000 nmol/L Verapamil (DAPI). α-actinin shows the sarcomere lengths aligned to the microgrooves. Similarly, as can be seen from Vinculin staining, the focal adhesion with the substrate is decreasing as the drug concentration increases. This data can be correlated with our impedance readouts, that depict decreasing cell index. In a cell, vinculin is connected to a talin filament, which in turn is attached to the integrin filament that adheres to the extracellular matrix [28]. On addition of Verapamil, the integrin expression decreases [29]. This subsequently leads to decrease of Vinculin expression and the total number of cells (DAPI).

**Figure 7:**
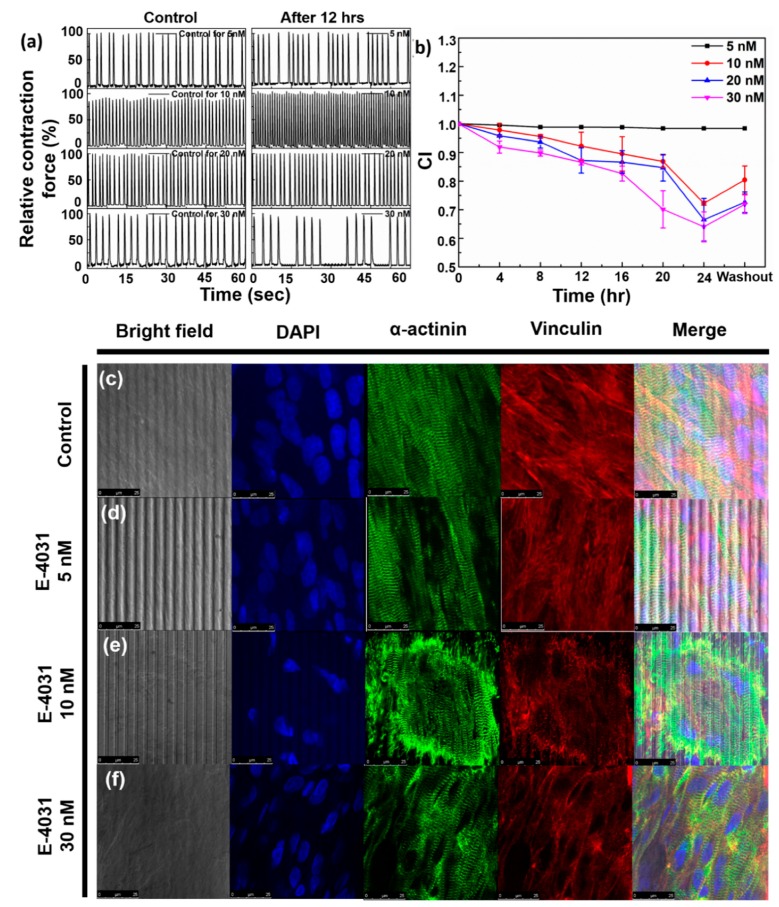
(**a**) Normalized contraction force on addition of E-4031 of concentrations 5 nmol/L, 10 nmol/L, 20 nmol/L, 30 nmol/L after 12 h, (**b**) Normalized impedance output on addition of E-4031 of concentrations 5 nmol/L, 10 nmol/L, 20 nmol/L, 30 nmol/L up to 24 h. (**c**–**f**) ICC staining data for (**c**) control, (**d**) 5 nmol/L, (**e**) 10 nmol/L, (**f**) 30 nmol/L E-4031. Scale bar = 25 µm.

Our device was also tested for its response with E-4031, which is a potassium ion channel blocker of the HERG channel and is known to cause prolongation of QT interval without any effect on contraction force. The contraction force of cardiomyocytes and their change in beating frequency along with impedance were measured up to 24 h for four different concentrations. Figure S6a–d show the relative change in contraction force measured up to 24 h for 5 nmol/L, 10 nmol/L, 20 nmol/L and 30 nmol/L concentrations respectively, and Figure 7a shows the relative contraction force after 12 h of addition of the drug of each concentration and the corresponding control state. It should be noted that Figure 7a shows contraction force data at 12 h from different samples. Hence, the beating frequency is different at each concentration. Figure 7b shows the cell index values up to 24 h. For concentrations of 5 nmol/L, 10 nmol/L and 20 nmol/L, although there is no significant change in beating frequency (Figure S6a–c), a decrease in cell index is observed. In case of E-4031, cell index decreases but does not recover, as was the case with Verapamil. In case of 10 nmol/L E-4031, CI value decreased up to 72% of its base value in 24 h and for 20 nmol/L, it was 66.5%. This shows that impedance methodology has the ability to sense cardiac toxicity even for small amounts of drug toxicity. For 30 nmol/L of E-4031, irregularities in beating patterns became prominent, as can be seen from Figure S6d. Beating frequency showed irregularities, that increased up to 12 h, after which it started to decrease. At 24 h, irregularities in beating frequency had further reduced but had not fully recovered. Figure S6e shows the overlapped curves of a single beat. QT interval was the longest when measured at 12 h, after which it started to decrease. At 24 h, QT interval had not fully recovered and was almost the same as that measured at 14 h. IC_50_ values of E-4031 vary from 3.4 nmol/L to 35 nmol/L, based on the assay method, above which significant change is observable [9]. In our case, changes were observable at 30 nmol/L concentration. Cell index also reduced up to 64% after 24 h (Figure 7b). However, cell index did not exhibit time-dependent recovery.

Figure 7c–f shows the ICC staining data for control, 5 nmol/L, 10 nmol/L and 30 nmol/L E-4031 to corroborate our obtained data. ICC staining experiments were performed 12 h after addition of drugs. The focal adhesion (Vinculin—red) decreases as compared to control. This result can be correlated to the decreasing cell index (Figure 7b). Number of cells (DAPI—blue) do not show significant change on increasing concentration. The sarcomere lengths (α-actinin—green) do not show significant difference. The focal adhesion reduces because E-4031 inhibits specific fibronectin-dependent cell adhesion [30]. On increasing the concentration of E-4031, cell adhesion decreases, as is evident from Vinculin data.

## 5. Conclusions

In this study, we have shown the working of a dual-function device with SU-8 as the base layer, which can simultaneously measure contraction force and electrophysiology of cardiomyocytes. The thicknesses of the SU-8 cantilevers were kept at 10 µm and 15 µm, and they were also aligned with microgrooves, so that the well-aligned cardiomyocytes can respond to the contraction force with a high cantilever deflection. The impedance readouts simultaneously provide information on growth of cells on the substrate. The data from cantilever deflection and impedance can be used to measure the response cardiac toxicity, i.e. its effect on contraction force and adhesion with the substrate. We tested this hypothesis with two drugs, namely Verapamil, which is a calcium ion channel blocker and E-4031, which is a potassium ion channel blocker. This device, however, is unable to provide the extracellular action potential data of the cells, which is another key factor for thorough analysis of cardiomyocytes. Hence, our future work will be focused on the fabrication of a device that can integrate all the key features of cardiomyocytes on a single platform.

## Supplementary Materials

The following are available online at https://www.mdpi.com/2072-666X/11/4/450/s1, Table S1: Details of dimensions of the IDE fabricated, Figure S1: (a) Optical images of the fabricated device (scale bar = 1 mm), (b) optical image of the microgrooves pattern on cantilever (c) optical image of type 1 IDE, (d) type 2 IDE, (e) type 3 IDE fabricated on the cantilever, Figure S2: (a) Optical image of the devices in cell culture medium, (b) bode plot of the impedance spectra of IDE types 1, 2 and 3, Figure S3: Measurement of impedance spectra at different cantilever displacements from 0 to 500 µm, Figure S4: Impedance spectra of base impedance and 3 days after cell seeding. Inset shows equivalent circuit between cell and substrate, Figure S5: Normalized contraction force on addition of Verapamil of concentrations (a) 150 nmol/L, (b) 300 nmol/L, (c) 500 nmol/L, (d) 1000 nmol/L measured up to 24 h, Figure S6: Normalized contraction force on addition of E-4031 of concentrations (a) 5 nmol/L, (b) 10 nmol/L, (c) 20 nmol/L, (d) 30 nmol/L measured up to 24 h, (e) overlapped peaks of contraction force of 30 nmol/L E-4031.
