# Supplementary material for: Polymer-Based Functional Cantilevers Integrated with Interdigitated Electrode Arrays—A Novel Platform for Cardiac Sensing
Source: Micromachines (Basel). 2020 Apr 24;11(4):450. doi: 10.3390/mi11040450 (PMC7231360; doi:10.3390/mi11040450)
Supplement: Supplementary file 1 [file micromachines-11-00450-s001.pdf]

Supplementary Materials: Polymer-Based Functional Cantilevers Integrated with Interdigitated Electrode Arrays—A Novel Platform for Cardiac Sensing

Pooja P. Kanade, Nomin-Erdene Oyunbaatar and Dong-Weon Lee

Table S1. Details of dimensions of the IDE fabricated.

IDE No.	No. of Fingers (N)	Finer Length (L µm)	Finger Width (W µm)	Spacing (S µm)	Electrode Length (mm)	K Value
Type 1	60	495	5	5	150	0.68
Type 2	30	990	10	10	297	0.70
Type 3	30	985	15	15	443	0.70

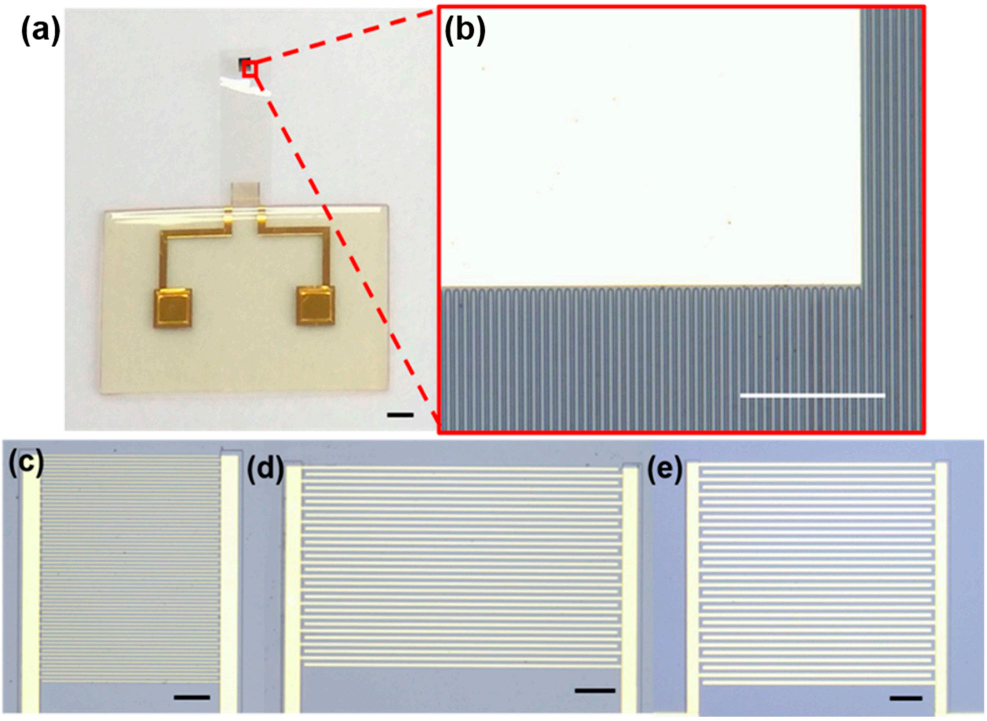

Figure S1. (**a**) Optical images of the fabricated device (scale bar = 1 mm), (**b**) optical image of the microgrooves pattern on cantilever (**c**) optical image of type 1 IDE, (**d**) type 2 IDE, (**e**) type 3 IDE fabricated on the cantilever (scale bars = 100 µm).

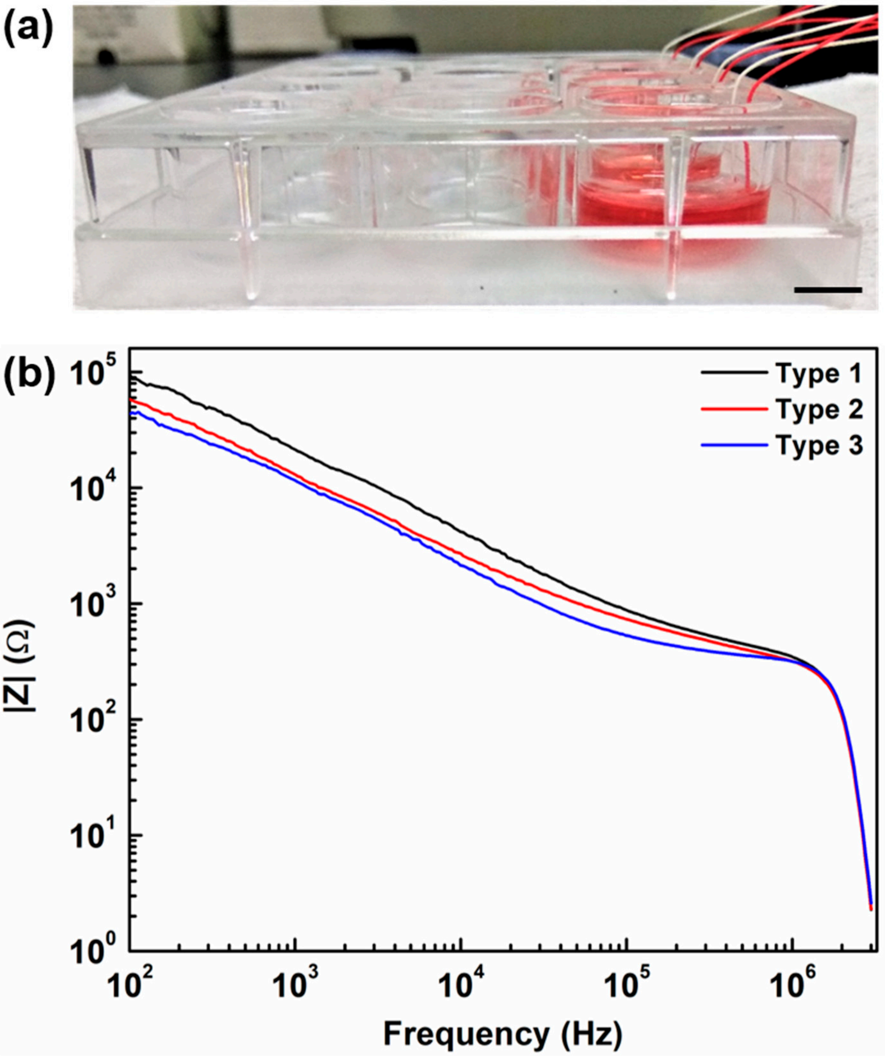

Figure S2. (**a**) Optical image of the devices in cell culture medium (scale bar = 10 mm), (**b**) bode plot of the impedance spectra of IDE types 1, 2 and 3.

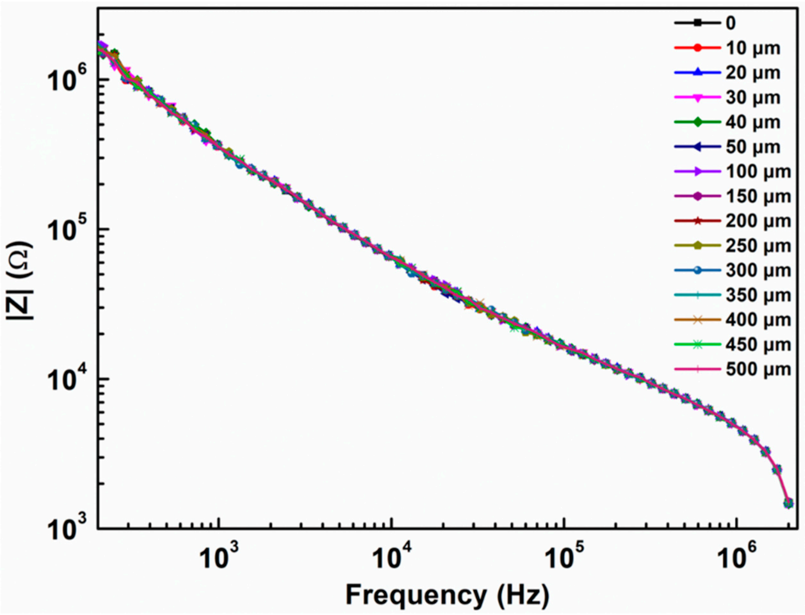

Figure S3. Measurement of impedance spectra at different cantilever displacements from 0 to 500 μm.

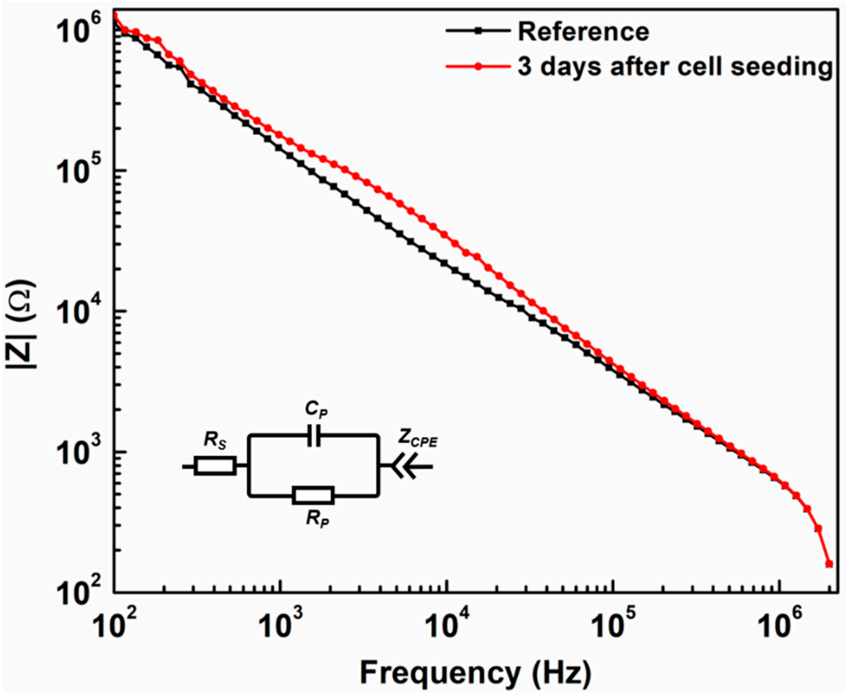

Figure S4. Impedance spectra of base impedance and 3 days after cell seeding. Inset shows equivalent circuit between cell and substrate.

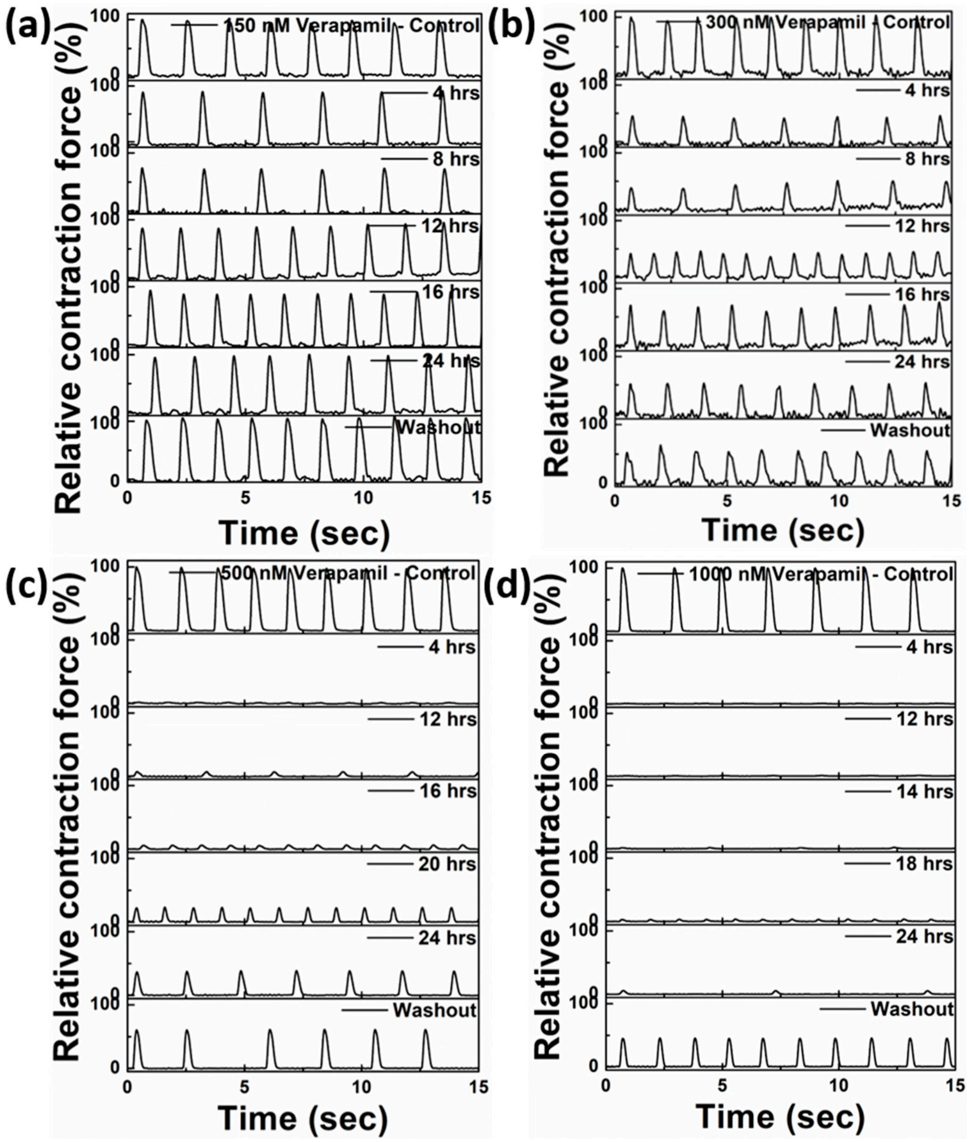

Figure S5. Normalized contraction force on addition of Verapamil of concentrations (**a**) 150 nmol/L, (**b**) 300 nmol/L, (**c**) 500 nmol/L, (**d**) 1000 nmol/L measured up to 24 h.

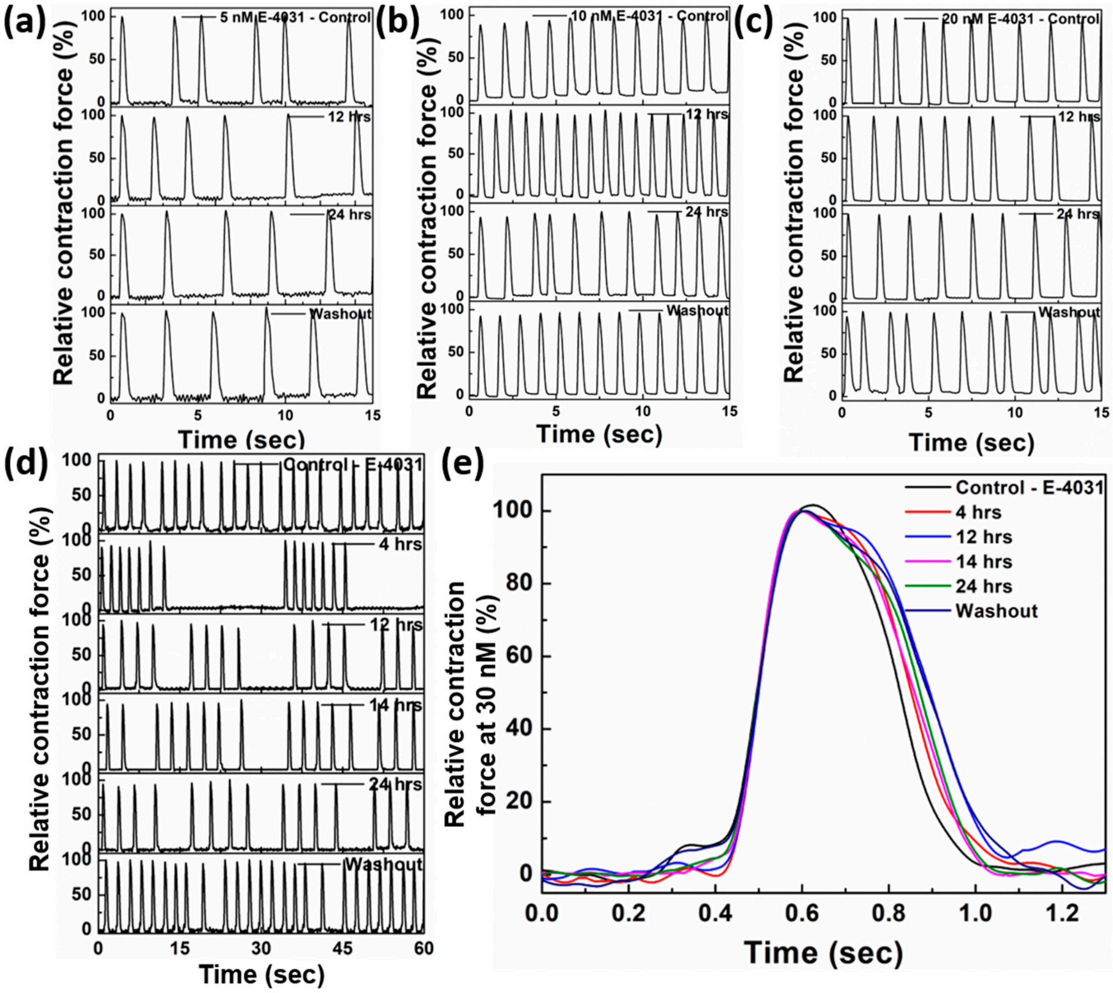

Figure S6. Normalized contraction force on addition of E-4031 of concentrations (**a**) 5 nmol/L, (**b**) 10 nmol/L, (**c**) 20 nmol/L, (**d**) 30 nmol/L measured up to 24 h, (**e**) overlapped peaks of contraction force of 30 nmol/L E-4031.
